# Aging-Accelerated Mouse Prone 8 (SAMP8) Mice Experiment and Network Pharmacological Analysis of Aged Liupao Tea Aqueous Extract in Delaying the Decline Changes of the Body

**DOI:** 10.3390/antiox12030685

**Published:** 2023-03-10

**Authors:** Wenjing Pan, Wangshu Li, Huan Wu, Xinya Xie, Mingwei Xie, Qing Nie, Zhonghua Liu, Shuxian Cai

**Affiliations:** 1 National Research Center of Engineering Technology for Utilization of Botanical Functional Ingredients, Hunan Agricultural University, Changsha 410128, China; 2 Key Laboratory of Ministry of Education for Tea Science, Hunan Agricultural University, Changsha 410128, China; 3 Co-Innovation Center of Education Ministry for Utilization of Botanical Functional Ingredients, Hunan Agricultural University, Changsha 410128, China; 4 Key Laboratory for Evaluation and Utilization of Gene Resources of Horticultural Crops, Ministry of Agriculture and Rural Affairs of China, Hunan Agricultural University, Changsha 410128, China; 5 Wuzhou Institute of Agricultural, Wuzhou 543003, China

**Keywords:** aged Liupao tea, aging-accelerated mouse prone 8 (SAMP8) mice, degenerative changes, network pharmacology, molecular docking

## Abstract

Aging and metabolic disorders feedback and promote each other and are closely related to the occurrence and development of cardiovascular disease, type 2 diabetes, neurodegeneration and other degenerative diseases. Liupao tea is a geographical indication product of Chinese dark tea, with a “red, concentrated, aged and mellow” flavor quality. In this study, the aqueous extract of aged Liupao tea (ALPT) administered by continuous gavage significantly inhibited the increase of visceral fat and damage to the intestinal–liver–microbial axis in high-fat modeling of SAMP8 (P8+HFD) mice. Its potential mechanism is that ALPT significantly inhibited the inflammation and aggregation formation pathway caused by P8+HFD, increased the abundance of short-chain fatty acid producing bacteria *Alistipes*, *Alloprevotella* and *Bacteroides*, and had a calorie restriction effect. The results of the whole target metabolome network pharmacological analysis showed that there were 139 potential active components in the ALPT aqueous extract, and the core targets of their actions were SRC, TP53, AKT1, MAPK3, VEGFA, EP300, EGFR, HSP90AA1, CASP3, etc. These target genes were mainly enriched in cancer, neurodegenerative diseases, glucose and lipid metabolism and other pathways of degenerative changes. Molecular docking further verified the reliability of network pharmacology. The above results indicate that Liupao tea can effectively delay the body’s degenerative changes through various mechanisms and multi-target effects. This study revealed that dark tea such as Liupao tea has significant drinking value in a modern and aging society.

## 1. Introduction

Aging and metabolic disorder-mediated degenerative changes promote the occurrence and development of chronic noninfectious diseases such as diabetes, cancer, Alzheimer’s disease, cardiovascular disease, etc. [1,2,3]. The aging of the body is often accompanied by an increase in oxidative stress and persistent chronic inflammation [4,5,6,7]. Oxidative stress contributes to the increase of glucose uptake and lipid synthesis and plays an essential role in the accumulation of cholesterol in the liver [8]. The accumulation of visceral fat secretes free fatty acids (FFA), interleukin-6 (IL-6), tumor necrosis factor-alpha (TNF-α) and angiotensinogen into the venous circulation [9], promoting the formation of heterotopic lipid deposition and degenerative diseases [10,11].

Liupao tea (Camellia sinensis (L.) O.kuntze) is one of the representative products of Chinese dark tea. It is initially produced in Liupao Town, Cangwu County, Wuzhou and Guangxi, and has the quality characteristics of red, concentrated, aged and mellow [12]. The standardized production process of Liupao tea includes 3 times piling up and color changing: wet piling up, dry piling up and aging, which is unique among dark tea processes [13]. The contents of phenolic acid, theabrownins, tea polysaccharides, cellulose and organic acids in Liupao tea increase with the aging and piling up process [14,15,16,17].

Liupao tea has a strong dispelling dampness effect. Modern pharmacology has proved that Liupao tea has antioxidant functions, regulating glycolipid metabolism, protecting the liver, enhancing immune function, promoting digestion, anti-coagulation, gastric emptying and intestine peristalsis [18,19,20,21]. Liupao tea reduces the release of free fatty acids, improves glucose uptake, inhibits insulin resistance and significantly protects cells [22]. Liupao tea shows a more robust iron-reducing antioxidant capacity than Fuzhuan Brick tea and Pu’er tea [23]. Liupao tea polyphenols can significantly improve the body’s antioxidant effects, inhibit the production of inflammatory factors such as IL-6, IL-12, TNF-α and IFN-γ and have an inhibitory effect on gastrointestinal damage, and its activity is similar to that of ranitidine [24,25]. Liupao tea increases the diversity of intestinal flora, the proportion of *Bacteroidota*/*Firmicutes* and the abundance of short-chain fatty acid (SCFAs)-producing species of *Prevotella* and *Bacteroidetes*, inhibits colonic inflammation and has protection against the hyperglycemia model mice effect [26,27,28].

Early age-related changes, such as hair loss and short life span, characterize senescence-accelerated mouse prone 8 (SAMP8) mice [29]. Rhea and Banks confirmed that SAMP8 mice can be used as animal models for studies of age-related emotion and memory dysfunction [30]. In recent years, network pharmacology has often been used to explore medicinal plants’ therapeutic effects and targets [31]. In this study, referring to the experimental study of Onishi et al., a model of lipid metabolism disorder in SAMP8 mice was established to study the in vivo experimental study of ALPT in delaying degenerative changes [32]. In addition, we used the widely targeted metabonomic data of ALPT water extract to conduct network pharmacological analysis with “degenerative changes” as the disease keyword.

## 2. Materials and Methods

### 2.1. Ethical Statement

All animals were handled following the guidelines of national animal care legislation. The Animal Protection and Utilization Committee of Peking University approved experimental procedures and protocols (SYXK(JING)2021-0064). All surgery was performed under sodium pentobarbital anesthesia, and efforts were made to minimize suffering.

### 2.2. Materials and Reagents

Seven-Year Aged Liupao Tea (ALPT) was presented by Guangxi Wuzhou Tea Factory Ltd. (Wuzhou, China). Metformin (Met) was ordered from Shanghai Biyuntian Biotechnology (Shanghai, China). Lipopolysaccharide (LPS) and indomethacin (Idm) were ordered from Sigma-Aldrich (St. Louis, MO, USA). Physiological saline and 75% ethanol were purchased from China Pharmaceutical Group Ltd. (Changsha, China). Paraformaldehyde and fat-specific fixative were purchased from Wuhan Seville Biotechnology Co, TC, TG, total antioxidant capacity (T-AOC), SOD and MDA kits were from Nanjing Jiancheng Institute of Biological Engineering (Nanjing, China). IL-6, ZO-1 and LPS ELISA kits were purchased from Jiang Sufia Biotechnology Co (Yancheng, China). In addition to anti-GAPDH, anti-NF-κB, anti-Histone H3, anti-AMPK, anti-p-AMPK, anti-mTOR, anti-p-mTOR, anti-Cyclin B1, anti-Cyclin D1 and anti-Sirt1 were also purchased (Cell Signaling, Boston, MA, USA). The following primary antibodies were also used for protein blotting analysis: anti-multiubiquitin (Medical & Biological Laboratories Co., Ltd, Tokyo, Japan) and anti-RAGE (Santa Cruz Biotechnology, San Cruise, CA, USA). Immobilon Western Chemiluminescent HRP Substrate was purchased from Millipore, Middlesex County, MA, USA, and all other reagents used were analytically pure or chromatographically pure.

### 2.3. Preparation of Tea Water Extract

In this experiment, the tea brewing and the direct freeze-drying methods were used with high fidelity to preserving the tea composition and particle structure in the tea soup. 100 g of tea leaves was accurately weighed into a conical flask filled with 1 L of 100 °C water, and then the conical flask was put into a 100 °C water bath for 45 min, filtered, and 1 L of 100 °C water was added again. The two tea soups were mixed, and after being cooled to room temperature, they were pre-frozen at −20 °C for 24 h and then freeze-dried in a vacuum freeze dryer (Alpha 1-4/LSC Plus, Christ, Osterode, Germany) at −42 °C for 24 h to obtain the tea extract, which was stored in bags at −20 °C.

### 2.4. Mice and Experimental Design

**Figure 1:**
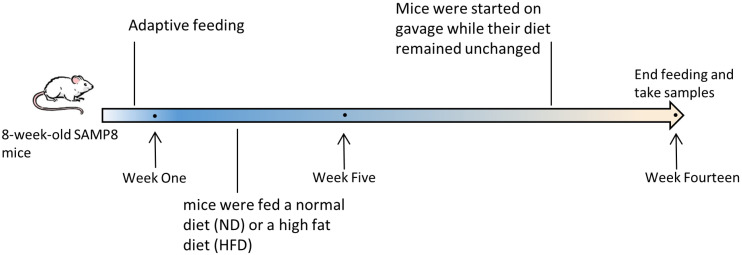
Experimental design in mice.

The 8-week-old male senescence-accelerated mouse prone 8 (SAMP8, P8) mice were purchased from Beijing Viton Lever Laboratory Animal Technology Co. After 1 week of adaptive feeding in SPF clean-grade animal rooms, P8 mice were randomly divided into 4 groups, namely the control group (P8+ND), high-fat modeling group (P8+HFD), metformin group (P8+HFD/Met) and aged Liupao tea group (P8+HFD/ALPT). P8+ND group mice were fed a basal diet, and the other groups were fed a 60% high-fat diet. After 4 weeks of high-fat modeling, the drug-treated groups were gavaged 200 mg/kg/d of Met and ALPT, respectively. The P8+ND and P8+HFD groups were given the same dose of drinking water, respectively, and the P8+HFD and drug-treated groups continued to eat the high-fat diet. After continuing treatment for 9 weeks, mice serum and tissues such as liver, epididymal fat, skin, small intestine and colon were taken to continue subsequent related experiments (Figure 1).

### 2.5. Paraffin Section Staining and Analysis

Mice tissues, such as liver, fat, skin and intestine, were immersed in fixative for 48 h, embedded in paraffin and prepared into 5 μm-thick sections. After hematoxylin-eosin (HE) staining, the sections were mounted with neutral gum. Finally, HE slices were photographed using an upright fluorescence microscope (Carl Zeiss/Axio Scope.A1) and morphological analysis and statistics were performed using Image J v1.8.0 software [33].

### 2.6. Biochemical Index Determination

We strictly followed the kit’s instructions, accurately weighed the liver tissue, added 9 times the volume of homogenization medium and mechanically ground it in a tissue grinder under ice-water bath conditions. After centrifugation, we took the supernatant and determined the content of TG, TC, T-AOC, SOD and MDA levels in liver tissue. The TC and TG levels in serum were detected strictly according to instructions [34].

### 2.7. FTIR Detection and Analysis

Liver tissue samples of different treatment groups were prepared by freeze-drying at −40 °C for 24 h using a vacuum freeze dryer (Christ, Germany). Two-hundred milligrams of potassium bromide and 2 mg of liver tissue samples were ground into tablets in an agate bowl to prepare infrared detection samples. A Fourier transform infrared spectrometer (FTIR) (PE-Spectrum 65, UK) was used to scan with 4000–400 cm^−1^ wavenumber range, 4 cm^−1^ resolution and accumulating 64 scans [35].

### 2.8. Western Blotting

Mice liver tissues were taken into RIPA lysis buffer containing a protease inhibitor, homogenized by an electric motor in an ice bath and centrifuged (4 °C, 12,000 rpm, 20 min) to collect the supernatant. A BCA protein quantification kit was used to determine the protein concentration of the sample to be tested. The loading buffer was added at a volume ratio of 1:4, and after boiling, the samples were subjected to SDS-PAGE electrophoresis by the equal mass loading method and then electrotransferred to PVDF membranes. PVDF membranes were blocked with TBST (Tris Buffered Saline with Tween) containing 5% skimmed milk for 1 h at room temperature and then incubated with primary antibody overnight at 4 °C. The membranes were washed 5~6 times with TBST for 5 min each time, incubated with horseradish peroxidase (HRP)-labeled secondary antibody for 90 min and then washed with TBST, followed by 1–2 min reaction with highly sensitive chemiluminescent substrate and exposed for 10 s to 2 min and finally, the protein bands were scanned in grayscale using Image J.

### 2.9. ELISA Assay

We strictly followed the ELISA kit’s instructions, accurately weighed the liver tissue and colon tissue, added 9 times the volume of RIPA lysate and mechanically ground the product in a tissue grinder under ice-water bath conditions. After centrifugation (4 °C, 12,000 rpm, 20 min), the supernatant was taken and LPS and IL-6 in liver tissue and ZO-1 in colon tissue were determined.

### 2.10. Detection and Analysis of Intestinal Flora 16S rRNA

The collected fresh fecal samples were immediately pre-frozen in liquid nitrogen for 2 min, frozen on dry ice and sent to Servicebio (Wuhan, China, https://www.servicebio.cn/) for 16S detection and analysis. According to the detection data provided by the company, the OUT was obtained by clustering the reads at a similarity level of 97% using Usearch software. Taxonomic annotation of the sequences using SILVA as a reference database gave the species classification information corresponding to each feature. Then, the community composition of each sample could be counted at each level (phylum, class, order, family, genus, species) and the abundance tables of species at different taxonomic levels were generated using QIIME software. QIIME and PICRUSt2 software analyzed the sample’s beta diversity and functional gene composition.

### 2.11. Widely Targeted Metabolomics Analysis of ALPT Aqueous Extract

The ALPT extract prepared in Method 2.3 was sent to Metware Biotechnology Co., Ltd. (Wuhan, China) for metabonomic analysis. We took 200 μL of the sample, added 200 μL of 70% methanol internal standard extract, vortexed for 3 min and centrifuged at 12,000 r/min, 4 °C for 10 min. The supernatant was removed and filtered through a microporous membrane (0.22 μm). The sample extracts were analyzed using a UPLC-ESI-MS/MS system (UPLC, ExionLC AD, https://sciex.com.cn/; MS, Applied Biosystems 6500 Q TRAP, https://sciex.com.cn/). The effluent was alternatively connected to an ESI-triple quadrupole linear ion trap (QTRAP)-MS. According to the secondary spectrum information, qualitative and quantitative analysis of substances was carried out in the Metware Database (MWDB) using triple quadrupole multiple reaction monitoring (MRM) mass spectrometry [36,37]. Metabolites whose secondary mass spectrometry (all fragment ions of the substances) and RT (retention time) information match the substance information in the database with a score of more than 0.5 were selected for subsequent analysis.

### 2.12. Screening of Active Metabolites

The compounds detected in metabolomics were converted into the Simplified Molecular Input Line Entry System (SMILES) through PubChem (https://pubchem.ncbi.nlm.nih.gov/ accessed on 16 November 2022). The SwissADME (http://www.swissadme.ch accessed on 24 November 2022) tool was used to predict potential active ingredients [38]. Gastrointestinal (GI) absorption was examined, and drug-likeness (DL) analysis was conducted. The parameters of GI meet “high” and at least meet the metabolites of two drug-likeness. It was selected as an active compound with good bioavailability.

### 2.13. Prediction of Active Ingredients and Disease Targets

With Probability* > 0.1 as the screening condition, the disease target of the active ingredient was predicted by Swiss Target Prediction (swisstargetprediction.ch accessed on 29 November 2022) [39]. We could obtain inflammation-related targets by retrieving the keyword “degenerative changes” from GeneCards. GeneCards is a database that integrates all annotation and prediction genes related to human diseases [40].

### 2.14. PPI Network and KEGG Enrichment Analysis

Venny 2.1 was used to analyze the active ingredient targets and genes related to degenerative changes, and the obtained common genes were introduced into the STRING v.11.5 (https://string-db.org/ accessed on 13 December 2022) analysis platform. Under the condition that the confidence score was more significant than 0.7, a protein-protein interaction network diagram (PPI) was obtained. Then, the compound target network was constructed and visualized using Cytoscape 3.9.1 and the topological importance, intermediate centrality and tight centrality of nodes in the network were analyzed. Finally, the Metascape (http://metascape.org accessed on 13 December 2022) analysis platform was used for the Kyoto Encyclopedia of Genes and Genomes (KEGG) enrichment analysis of common genes [41,42].

### 2.15. Molecular Docking

Autodock software 4.2 was used to perform protein compound docking analysis [43]. Firstly, three-dimensional (3D) molecular structures of target proteins and ligand compounds were downloaded from the Protein Data Bank database (http://www.rcsb.org/ accessed on 24 December 2022) and PubChem (https://pubchem.ncbi.nlm.nih.gov/ 24 December 2022) database. Next, PyMOL and AutoDock Tools 1.5.7 were used for protein and ligand pre-processing, including removal of original water ligands, hydrogenation, calculation of charge numbers, atomic AD4 type assignment and detection of torsional bonds. Finally, the grid box was set to the entire region and the Genetic Algorithm was selected for docking in AutoDock Tools 1.5.7. Finally, the docking results with the lowest binding energy were visualized using PyMOL software.

### 2.16. Data Processing and Analysis

Data analysis was performed using GraphPad Prism version 8.0. One-way ANOVA and Tukey’s multiple comparison tests were used to analyze the significance of differences. The results were expressed as mean ± standard deviation and the difference was judged to be significant according to *p* < 0.05 and *p* < 0.01.

## 3. Results

### 3.1. Aged Liupao Tea (ALPT) Delays the Degenerative Changes of SAMP8 High-Fat Diet Mice

#### 3.1.1. ALPT Maintains Metabolic Homeostasis

**Figure 2:**
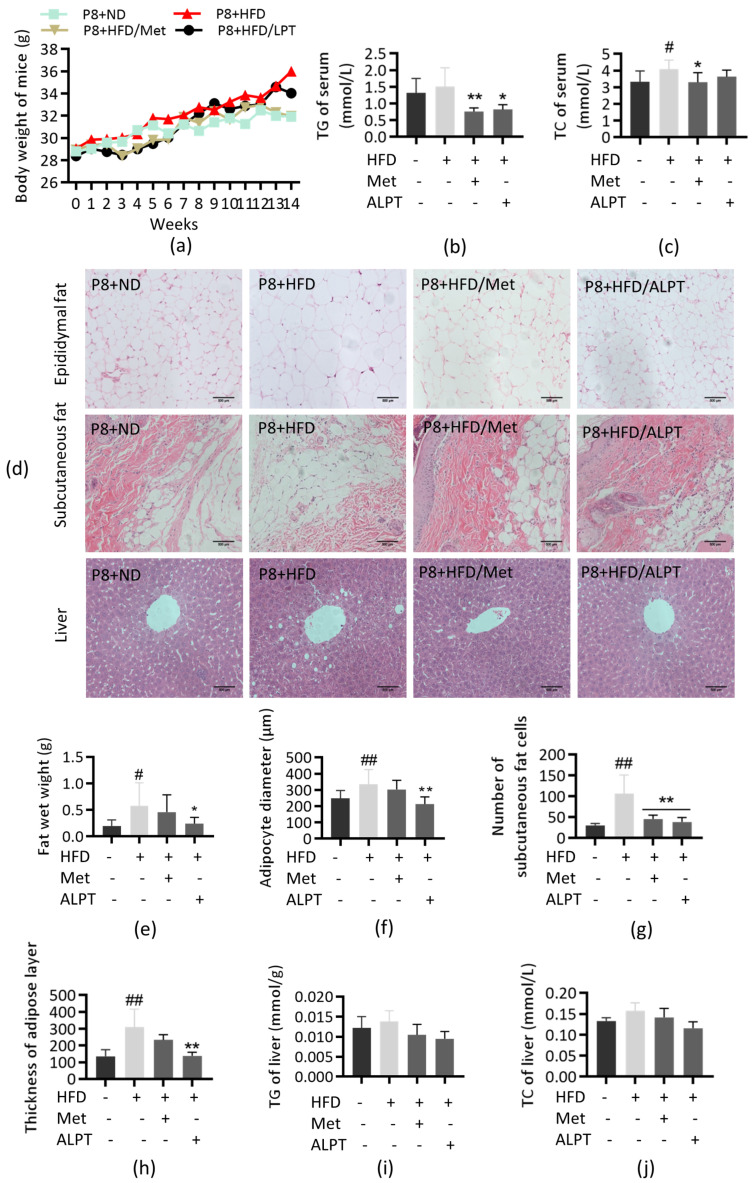
ALPT improved lipid metabolism in high-fat diet SAMP8 mice. (**a**) A body weight measurement of mice in different treatment groups, n = 10; (**b**,**c**) determination of serum triglyceride (TG) and total cholesterol (TC) content, n = 10; (**d**) HE staining of epididymal fat, skin and liver (200×); (**e**,**f**) wet weight and cell diameter of epididymal fat, n = 5; (**g**,**h**) measurement of the thickness of the subcutaneous fat layer, n = 5; (**i**,**j**) liver TG and TC content test, n = 5. # represents compared with P8+ND group, *p* < 0.05, ## represents compared with P8+ND group, *p* < 0.01, * represents compared with P8+HFD group, *p* < 0.05, ** represents compared with P8+HFD group, *p* < 0.01.

Body weight measurement data showed that mice in the P8+HFD and P8+HFD/ALPT groups had higher body weight after 14 weeks of feeding (Figure 2a). The kit assay results of cholesterol (TC) and triglyceride (TG) showed that the HFD diet significantly increased the levels of serum TC and TG in P8 mice, while ALPT had an inhibitory effect (Figure 2b,c). HE staining showed that in the P8+HFD group, the epididymal adipocytes were hypertrophied (*p* < 0.05) (Figure 2d–f), the skin structures were disordered, the number of subcutaneous adipocytes and the thickness of the fat layer were significantly increased and the boundaries between subcutaneous muscle and adipocytes were blurred (Figure 2d,g,h). Met and ALPT significantly reduced fat accumulation (*p* < 0.05 or *p* < 0.01). In addition, the hepatic sinusoids of P8+HFD group mice were unclear, the cells were arranged disorderly, the nuclei were deeply stained and many fat droplets were accumulated around the manifolds and the contents of TC and TG were significantly increased. In the P8+HFD/ALPT group, the hepatocytes were clear, arranged radially, without inflammatory cell infiltration (Figure 2d), lipid droplets decreased and TC and TG contents decreased (Figure 2i,j).

#### 3.1.2. ALPT Delays Liver Aging

**Figure 3:**
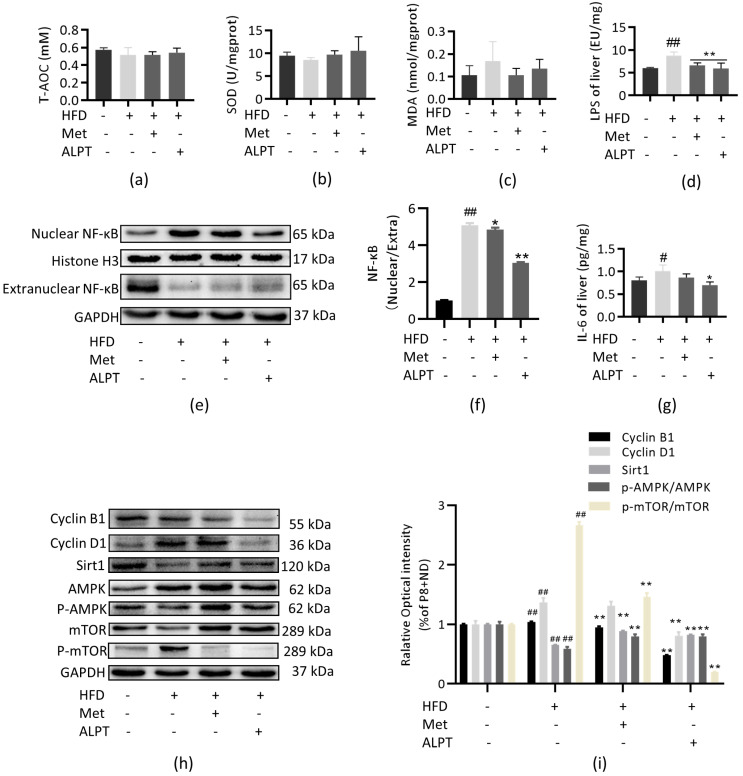
ALPT delays liver aging by anti-inflammatory and metabolic regulation. (**a**–**c**) Total antioxidant capacity (T-AOC), superoxide dismutase (SOD) and malondialdehyde (MDA) content assay, n = 5; (**d**,**g**) lipopolysaccharide (LPS) and interleukin-6 (IL-6) content assay, n = 5; (**e**,**f**) western blotting detection of nuclear transcription factor-kappa B (NF-κB), n = 3; (**h**,**i**) western blotting detection of G2/M-specific cyclin B1 (Cyclin B1), G1/S-specific cyclin D1 (Cyclin D1), Sirtuin 1 (Sirt1), Adenosine 5′-monophosphate (AMP)-activated protein kinase (AMPK), mammalian target of rapamycin (mTOR), phospho-adenosine 5′-monophosphate (AMP)-activated protein kinase (p-AMPK) and phospho-mammalian target of rapamycin (p-mTOR), n = 3. # represents compared with P8+ND group, *p* < 0.05, ## represents compared with P8+ND group, *p* < 0.01, * represents compared with P8+HFD group, *p* < 0.05, ** represents compared with P8+HFD group, *p* < 0.01.

The results of oxidative stress-related indexes showed that the content of MDA, TC and TG in the P8+HFD group increased significantly, while the contents of T-AOC and SOD decreased; both Met and ALPT inhibited the increase of P8+HFD-induced oxidative stress (Figure 3a–c).

The test results of inflammation-related indicators showed that compared with the P8+ND group, the contents of LPS and IL6 in the livers of P8+HFD group mice were significantly increased (*p* < 0.01 or *p* < 0.05) and the nuclear transfer of NF-κB increased about 6 times (*p* < 0.01). ALPT significantly inhibited P8+HFD-induced inflammation, and its activity was superior to Met (Figure 3d–g).

The results of protein expression detection related to aging, cycle and metabolism showed that, compared with P8+ND group, the expression of Cyclin D1 and Cyclin B1 in the P8+HFD group was upregulated (*p* < 0.01) and the expression of Sirt1 was downregulated (*p* < 0.01); the phosphorylation level of AMPK decreased (*p* < 0.01) and the phosphorylation level of mTOR increased about 2.8-fold (*p* < 0.01). ALPT significantly inhibited P8+HFD-induced signaling pathway changes (Figure 3h,i). The above results indicate that ALPT can inhibit liver aging and metabolic disorders in the P8+HFD group.

**Figure 4:**
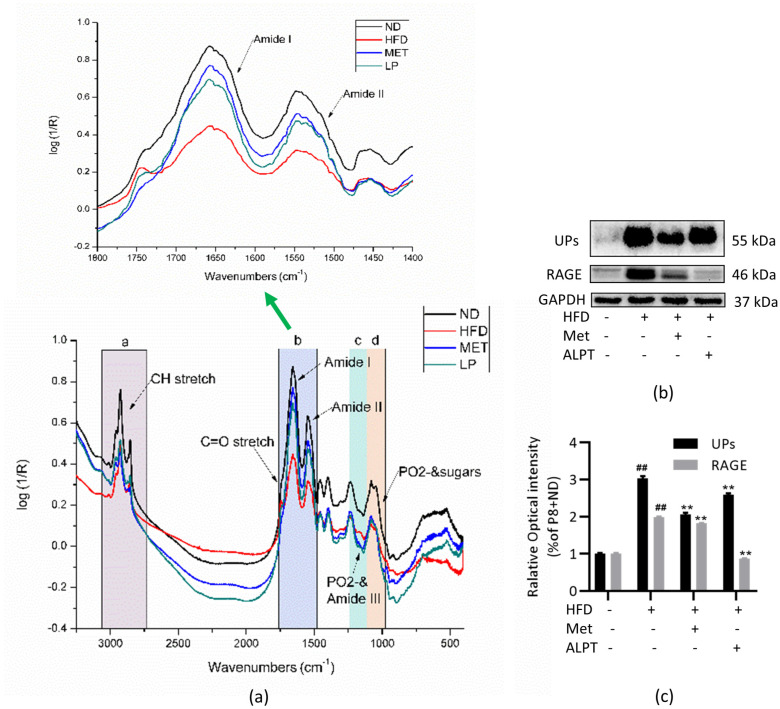
APLT inhibits P8+HFD-induced aggregates formation pathway and biomacromolecule structure alterations. (**a**) Infrared spectra of the liver. (**b**,**c**) Western blotting detection of ubiquitin-conjugated proteins (Ups) and receptor for advanced glycation end products (RAGE), n = 3. In the infrared (IR) spectrum, the absorption band of 3015–2800 cm^−1^ mainly characterizes the C-H bond of long-chain fatty acids and the intensity of this region is closely related to the lipid content [35,51]; 3012 cm^−1^ characterizes the ν=C-H bond of unsaturated fatty acids [52] and 1740 cm^−1^ is stretching vibrations of C=O groups of lipids [53]; 1700–1500 cm^−1^ is the protein amide I and II bands’ absorption peaks [54]; 1300–1000 cm^−1^ is the absorption band of the nucleic acid structure [55]. ## represents compared with P8+ND group, *p* < 0.01, ** represents compared with P8+HFD group, *p* < 0.01.

With the growth of age, proteins, lipids, nucleic acids and other biological molecules react with ROS, resulting in many carbonylation-modified biological molecules stored in the body, which can be used as one of the biomarkers of body aging [44,45,46]. The absorption of amide I contains the contribution of C=O stretching vibration from the amide group (about 80%). The stronger the hydrogen bond involving amide C=O, the lower the electron density in the C=O group and the lower the absorption peak of amide I and amide II bands [47]. The results of Fourier infrared spectrometer (FTIR) analysis showed that, compared with the P8+ND group, the fatty acid absorption peak (3015–2800 cm^−1^), protein peak (1700–1500 cm^−1^), lipid C=O groups (1740 cm^−1^) and nucleic acid peak (1300–1000 cm^−1^) of P8+HFD group shifted to the lower wavelength and the peak intensity decreased. The amide I and amide II bands (1700–1500 cm^−1^) were enlarged (Figure 4a, the figure shown by green arrow). Compared with the P8+ND group, the absorption peak intensity of the P8+HFD group decreased significantly and shifted to a lower wavenumber. The IR peaks in P8+HFD/Met and P8+HFD/ALPT groups were similar to those in the P8+ND groups (Figure 4a).

Aging and metabolic disorders damage the ubiquitin–proteasome hydrolysis system, accumulate ubiquitinated modified protein aggregates and further mediate oxidative stress and inflammatory responses [48]. RAGE is a product of non-enzymatic glycosylation and protein and lipid oxidation. RAGE activation by various ligands enhances oxidative stress, activates the nuclear factor-κB (NF-κB) signaling pathway and induces inflammation [49,50]. Compared with the control group, the ubiquitin conjugated proteins (UPs) modified proteins in the liver of P8+HFD group mice increased and the expression level of RAGE was upregulated (*p* < 0.01), while Met and ALPT had significant inhibitory effects (*p* < 0.01) (Figure 4b,c). The results showed that ALPT significantly reduced the aggregates formation pathway and maintained the fidelity of biomacromolecules.

#### 3.1.3. Role of ALPT in Protecting Intestinal Homeostasis

**Figure 5:**
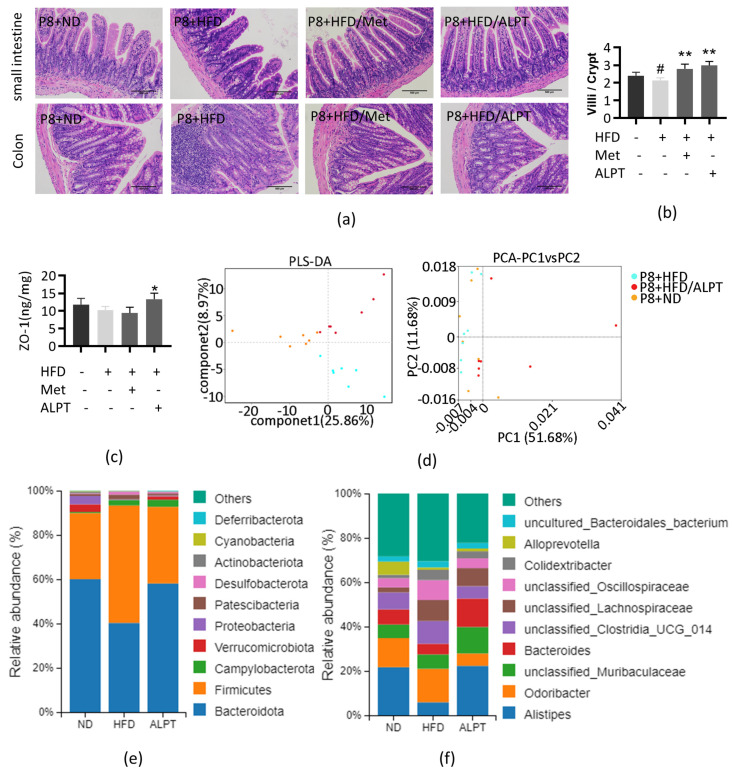
Analysis of intestinal structure and intestinal flora composition of mice in different groups. (**a**) HE staining of small intestine and colon (200×); (**b**) statistical analysis of the ratio of villus length to crypt depth (V/C); (**c**) colon connexin ZO-1 content analysis, n = 5; (**d**) PLS-DA and PCA principal coordinate analysis; (**e**) analysis of intestinal flora phylum classification; (**f**) analysis of intestinal flora genus classification. # represents compared with P8+ND group, *p* < 0.05, * represents compared with P8+HFD group, *p* < 0.05, ** represents compared with P8+HFD group, *p* < 0.01.

The intestinal HE staining results showed that compared with the P8+ND group, the intestinal villus structure of P8+HFD group mice was atrophied and damaged, the ratio of villus length to crypt depth (V/C) was significantly reduced, the intestinal epithelial cells were arranged irregularly and the nuclei were concentrated and stained deeply. In addition, P8+HFD led to a decrease in colonic goblet cells and infiltration of inflammatory cells. Met and ALPT treatment could significantly inhibit P8+HFD-induced intestinal homeostasis disorder (Figure 5a,b).

Zonula occludens 1 (ZO-1) can maintain cell polarity and play a crucial role in intestinal epithelial barrier integrity [56]. The ELISA assay showed that the ZO-1 protein content in the colon of P8+HFD group mice was lower than that of the P8+ND group, and the ZO-1 protein content in P8+HFD/ALPT group increased (*p* < 0.05), even higher than that of the P8+ND group (Figure 5c).

The composition of the intestinal microbiota was compared through β-diversity analysis. The principal coordinate analysis (PCA) and partial least squares discriminant analysis (PLS-DA) showed that the P8+HFD/ALPT group was far away from the P8+HFD group and close to the P8+ND group. The results showed that ALPT could inhibit P8+HFD-induced changes in intestinal flora composition (Figure 5d).

At the level of phylum classification, compared with the P8+ND group, P8+HFD group mice had a higher abundance of *Firmicutes* (F) and a lower abundance of *Bacteroidota* (B), that is, the F/B radio increased. Compared with the P8+HFD group, the F/B ratio in P8+HFD/ALPT group decreased (Figure 5e). At the level of genus classification, the abundance of *Alistipes*, *Alloprevotella* and *Bacteroides* producing short-chain fatty acid in the P8+HFD group decreased; the abundance of *Alistipes* and *Bacteroides* in the P8+HFD/ALPT group increased significantly, even higher than that in the P8+ND group (Figure 5f).

### 3.2. Widely Targeted Metabolome of ALPT Aqueous Extract and Its Network Pharmacological Analysis

The results of the multi-peak detection plot of metabolites in the MRM (Figure S1a,b) showed that the total ion flow curve of metabolite detection had high overlap; that is, the retention time and peak intensity were consistent, indicating that the instrument signal stability was good.

**Figure 6:**
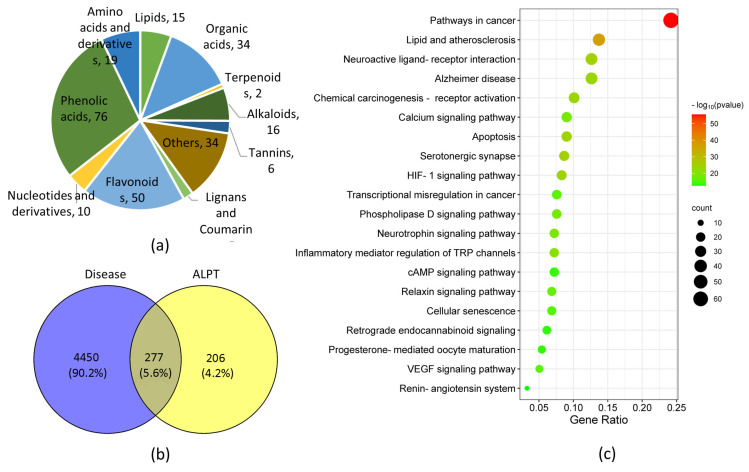
Widely targeted metabolomics of ALPT aqueous extract and their network pharmacological analysis. (**a**) Classification diagram of metabolites of ALPT aqueous extract; The numbers in the pie chart represent the number of metabolites. (**b**) Venn analysis was used to screen the target genes of ALPT water extract metabolites delaying degenerative changes; (**c**) KEGG analysis of target genes of ALPT water extract metabolites delaying degenerative changes.

The detection results of UPLC-ESI-MS/MS extensively targeted metabolites showed that 267 metabolites were detected in ALPT aqueous extract. These components were mainly phenolic acids, flavonoids, organic acids, alkaloids, amino acids and derivatives and lipids (Figure 6a). These compounds have metabolic, anti-inflammatory, antioxidant, anti-aging and other activities related to delaying degenerative changes [57,58,59].

According to the screening conditions of “suitable oral availability” and “drug-like values”, combined with literature research, 139 active metabolites (Table S1) were selected from 267 metabolites of ALPT aqueous extracts for subsequent network pharmacological analysis.

The analysis results of Gene Card and Swiss Target Prediction databases showed that there were 483 target genes in 139 active metabolites of ALPT aqueous extract. A total of 4727 target genes were screened with “degenerative changes” as the key work of the disease. Venn analysis showed 277 cross-genes between them (Figure 6b).

The results of KEGG analysis showed that the target genes related to the degenerative changes in the action of the active components of ALPT water extract were mainly enriched in cancer pathways, lipid atherosclerosis, Alzheimer’s disease, the calcium signaling pathway, the neurotrophic protein signaling pathway, inflammatory mediator regulation of TRP channel, cell aging, VEGF and other signaling pathways (Figure 6c). These pathways are closely related to cancer, neurodegenerative diseases, aging, glucose and lipid metabolism, energy metabolism and other degenerative changes.

**Figure 7:**
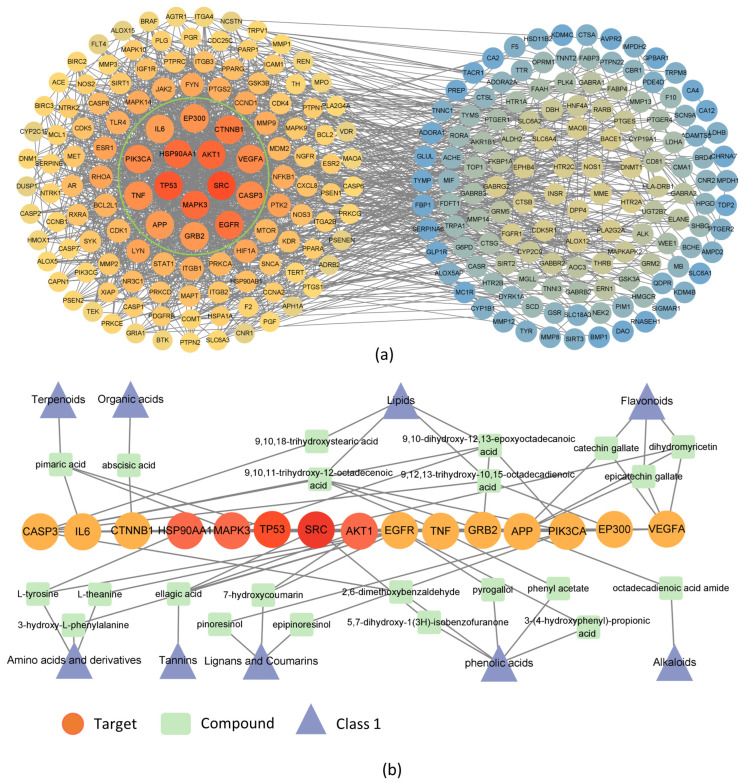
Network pharmacological analysis of active components of ALPT water extract and degenerative changes in the body. (**a**) PPI network analysis of the crossing genes. The genes in the PPI map on the left were the nodes whose degree value is greater than the median value, and those on the right were the nodes whose degree value is less than the median value; The more yellow the color, the greater the degree value. (**b**) The association analysis of the active substances and the most significant 15 target genes in the PPI network diagram.

PPI analysis showed many genes related to degenerative changes in the action of ALPT active components. We selected the node genes whose protein interaction is greater than the median value of PPI network mapping for visualization mapping (Figure 7a). The essential target genes of ALPT were SRC, TP53 and AKT1, respectively, MAPK3, HSP90AA1 and EGFR, etc. The results of correlation analysis showed that the active components of ALPT aqueous extract, such as dihydromyricetin, 5,7-dihydroxy-1(3H)-isobenzofuranone, pimaric acid, L-theanine, 7-hydroxycoumarin, ellagic acid and L-tyrosine, interacted with their target genes (Figure 7b).

**Figure 8:**
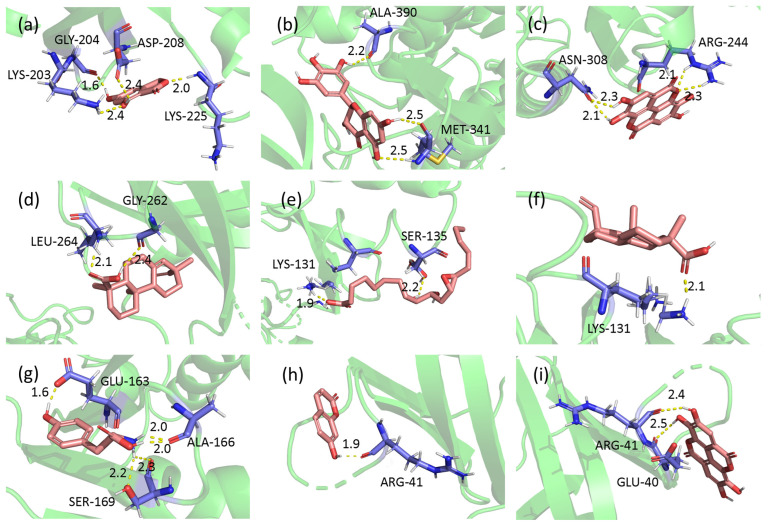
Docking conformation of five key target proteins and their combined components. (**a**) SRC to 5,7-Dihydroxy-1(3H)-isobenzofuranone. The binding energy is −3.481. (**b**) SRC to dihydromyricetin. The binding energy is −2.17. (**c**) SRC to ellagic acid. The binding energy is −3.47. (**d**) TP53 to pimaric acid. The binding energy is −5.83. (**e**) MAPK3 to 9,10-Dihydroxy-12,13-epoxyoctadecanoic acid. The binding energy is −0.47. (**f**) MAPK3 to pimaric acid. The binding energy is −5.47. (**g**) HSP90AA1 to L-tyrosine. The binding energy is −3.76. (**h**) AKT1 to 7-hydroxycoumarin. The binding energy is −5.28. (**i**) AKT1 to ellagic acid. The binding energy is −5.17.

The results of network pharmacology were further verified by molecular docking. The five most essential target proteins of ALPT and their associated active components were selected for molecular docking. The results showed the binding energies of TP53 with pimaric acid, MAPK3 with pimaric acid, AKT1 with 7-hydroxycoumarin and AKT1 with ellagic acid were lower than −5 (Figure 8).

## 4. Discussion

In this study, we explored the activity and mechanism of ALPT in delaying the aging change through the data of mice in an in vivo experiment and network pharmacology. Literature data showed that SAMP8 mice had senescence phenotype at 4 months [60,61]. At the end of this study, SAMP8 mice reached 5.5 months of age. P8+HFD mice showed lipid metabolism disorder, liver aging, intestinal structure damage and intestinal bacterial composition change. ALPT protected the liver intestine microbial axis of P8+HFD mice and had significant metabolic regulation and anti-aging effects.

### 4.1. ALPT Protects the Intestinal–Liver–Microbial Axis

Aging, a high-fat diet and intestinal flora disorders promote and feedback each other [62,63]. With the increase of the ratio of villus length to crypt depth (V/C), the contact surface with food increases, indicating that the small intestinal function is better [64,65]. ALPT inhibited P8+HFD induced the decrease of V/C in the small intestine and the number of goblet cells in the colon (Figure 4a,b). Colonic goblet cells can secrete a high molecular weight glycoprotein called mucin to protect the intestinal epithelium from physical damage caused by lumen contents, prevent bacterial invasion and interact with immunoglobulin A to play the role of antibody and antitoxin [66,67]. ALPT significantly increased the ZO-1 protein content of P8+HFD mice and protected intestinal mucosa (Figure 5c).

The ratio of *Firmicutes* to *Bacteroidota* (F/B) is an important indicator to describe the structure of intestinal flora. The high-fat diet promotes the production of LPS and increases the F/B ratio [68,69,70]. Clinical studies have shown that the abundance of *Bacteroidota* in the intestinal bacteria of the elderly is reduced [71,72]. ALPT inhibited the increase of the F/B ratio of HFD+P8 mice and the decrease of the abundance of short-chain fatty acid-producing bacteria, *Alistipes*, *Alloprevotella* and *Bacteroides* (Figure 5d–f). Butyric acid produced by *Alistipes* can also increase the integrity of intestinal epithelial tight junction-mediated barriers [73].

LPS is an endotoxin produced by pathogenic microorganisms. Studies have shown that when the intestinal barrier function decreases, LPS enters the blood through intestine leakage and then flows into the liver, leading to liver damage and promoting visceral fat accumulation [74]. Pathological section and biochemical analysis showed that P8+HFD aggravated intestinal inflammation, increased LPS content, decreased ZO-1 protein expression and increased intestinal permeability. Inflammation, endotoxin and fat accumulation aggravate liver injury. ALPT significantly protected the structure and function of the intestinal–liver–microbial axis in P8+HFD mice.

### 4.2. Metabolic Regulation of ALPT

Aging is associated with loss of fat-free mass (primarily skeletal muscle) and increased fat mass [75]. In this study, both P8+HFD and P8+HFD/ALPT mice gained weight, but the visceral fat content of P8+HFD/ALPT mice was significantly lower than that of P8+HFD mice, showing significant differences in health status. In the P8+HFD group, the levels of TG and TC in serum were significantly increased, and intestinal tissue disorder and liver steatosis were observed. The liver is a central organ that controls lipid homeostasis through biochemical, signaling and cellular pathways. It plays a key and important role in lipid metabolism and has a huge capacity for lipid accumulation [76,77]. ALPT inhibited P8+HFD-induced heterotopic lipid deposition in the liver (Figure 2).

Short-chain fatty acids (SCFAs) are colonic anaerobes’ final products of carbohydrate degradation. Colonic anaerobes can degrade indigestible food and complex carbohydrates, change the PH value of the colon, provide a source of cellular energy, inhibit inflammation and promote metabolism [78]. ALPT increased the abundance of SCFAs-producing bacteria such as *Alistipes*, *Alloprevotella* and *Bacteroides* in the intestine of P8+HFD mice (Figure 5f). *Alistipes* mainly produce butyric acid in the intestine. Butyrate is essential in differentiating colonic T regulatory cells and activating intestinal and blood antigen-producing cells (APC) and T cells [79]. Butyrate is the energy source of colon cells and triggers the reduction of dietary energy harvesting in *Firmicutes*, thereby reducing fat accumulation in adipose tissue in the host [80,81]. Therefore, butyrate is one of the most important components in SCFAs. *Alloprevotella* and *Bacteroides* have glycolytic activity and produce appropriate acetic acid [82,83], which can attenuate colitis induced by high sugar [84]. In addition, *Alloprevotella* is an anti-inflammatory bacterium negatively related to LDL cholesterol [85]. This study further proved the effects of Liupao tea on improving body metabolism from the perspective of improving intestinal bacteria composition.

### 4.3. ALPT Delays Aging through Anti-Inflammatory and Caloric Restriction

Chronic inflammation is mediated by aging and metabolic disorders, promotes hyperfunction and mediates the occurrence and development of metabolic diseases such as diabetes, cardiovascular diseases and neurodegenerative diseases [86,87,88,89,90]. Previous studies have shown that dark tea has more antioxidant and anti-inflammatory effects than green tea in vivo. In addition, it has a significant role in regulating lipid metabolism [91]. Oxidative stress can cause the oxidation of polyunsaturated fatty acids, form lipid hydroperoxides [92], damage protein hydrolysis and induce the accumulation and aggregation of damaged or abnormal proteins in cells [93]. ALPT reduced oxidative stress, UPs-modified proteins and RAGE aggregation pathway in the liver of P8+HFD mice, and protected proteins, fatty acids, lipids, nucleic acids and other protein macromolecules in the liver. At the same time, ALPT significantly inhibited P8+HFD-induced NF-κB nuclear translocation and IL-6 section (Figure 3 and Figure 4).

In the process of organism aging, cell hyperfunction and signal feedback blocking are inhibited by caloric restriction (CR), so that cells are in a resting state (quiescence), which is the key to achieving healthy aging [94,95,96]. At this time, the cell cycle block is conducive to maintaining cells in a young state [97]. In P8+HFD model mice, ALPT inhibited the expression of cyclin B1 and cyclin D1, activated the Sirt1/AMPK pathway and decreased phosphorylation of mTOR expression. The results indicate that ALPT has a caloric restriction effect (Figure 3).

### 4.4. Correlation Analysis between Active Components of ALPT Aqueous Extract and Targets of Degenerative Changes

Fermented tea such as dark tea contains more abundant oxidized polyphenols, theophylline, polysaccharides, ellagic acid and fatty acids, which can target to regulate AMPK and G-protein coupled receptors to promote lipid metabolism and delay degenerative diseases [98,99,100]. Liupao tea has the characteristics of red, thick, aged and mellow flavor quality. Its three unique discoloration processes, wet billet accumulation, dry billet accumulation and aging make Liupao tea contain more soluble sugar, total free amino acids and theabrownins [13]. These ingredients have significant antioxidant, anti-inflammatory, metabolic regulation and liver protection effects [101].

ALPT aqueous extract was rich in oxidized polyphenols, phenolic acids, fatty acids and other components (Figure 6a). The gene targets of these components mainly focused on cancer pathways, the interaction of neuroactive ligand–receptor interactions, lipid and atherosclerosis, Alzheimer’s disease and other pathways (Figure 6c). The association analysis between the core target genes and the active ingredients of ALPT was shown in Figure 7 and Figure 8, and some results had been proved by relevant research. For example, 7-hydroxycoumarin upregulates protein kinase B (Akt) signaling, inhibits the level of inflammatory cytokines and has neuroprotective effects [102]. Ellagic acid and L-theanine have been shown to reduce EGFR expression and phosphorylation and inhibit tumor growth [103,104]. Pimaric acid can significantly inhibit the secretion of IL6 by LPS-stimulated RAW 264.7 macrophages [105].

## 5. Conclusions

In conclusion, the aqueous extract of aged Liupao tea (ALPT) has the effect of delaying aging-related degenerative changes in the body through anti-inflammation, caloric restriction, protection of the gut–liver axis and multi-targeting. Tea is rich in secondary metabolites beneficial to human health, such as polyphenols, pigments, polysaccharides, alkaloids, free amino acids and saponins. Current research shows that drinking tea is safe for the human body [101]. A growing number of studies have proved that dark tea has more significant in vivo effects than green tea in delaying aging and regulating metabolism. Tea processing can reduce toxicity and improve therapeutic efficacy, just like the processing of traditional Chinese medicine [106]. This study systematically investigated the efficacy and mechanism of Liupao tea in delaying degenerative changes, providing a theoretical basis for the drinking value of Liupao tea and other dark teas in a modern aging society.

## Data Availability

Data are contained within the article and Supplementary Materials.

## Supplementary Materials

The following supporting information can be downloaded at: https://www.mdpi.com/article/10.3390/antiox12030685/s1, Supplementary File.
