# Supplementary material for: Aging-Accelerated Mouse Prone 8 (SAMP8) Mice Experiment and Network Pharmacological Analysis of Aged Liupao Tea Aqueous Extract in Delaying the Decline Changes of the Body
Source: Antioxidants (Basel). 2023 Mar 10;12(3):685. doi: 10.3390/antiox12030685 (PMC10045736; doi:10.3390/antiox12030685)
Supplement: Supplementary file 1 [file antioxidants-12-00685-s001.zip › supplementary file/Figure S1/Description.pdf]

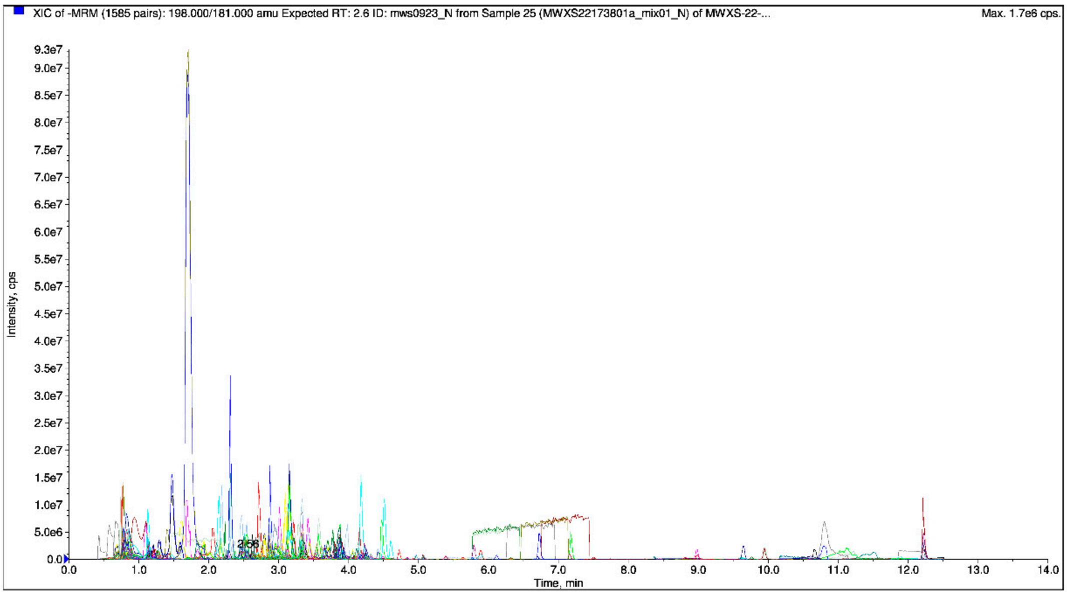

(a)

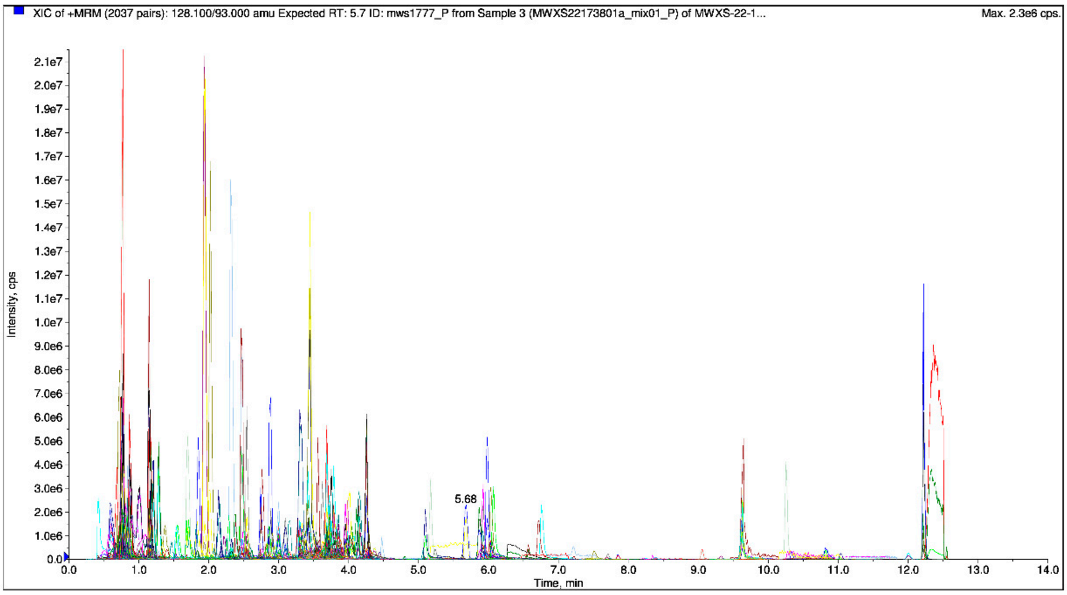

(b)

Figure S1. (a, b) multi-peak detection plot of metabolites in the multiple reaction monitoring mode of tea QC samples detected by mass spectrometry. Note: (a) is a negative ion mode, (b) is a positive ion mode and each mass spectrum peak with different colors represents a metabolite detected.
