# Supplementary material for: Aging-Accelerated Mouse Prone 8 (SAMP8) Mice Experiment and Network Pharmacological Analysis of Aged Liupao Tea Aqueous Extract in Delaying the Decline Changes of the Body
Source: Antioxidants (Basel). 2023 Mar 10;12(3):685. doi: 10.3390/antiox12030685 (PMC10045736; doi:10.3390/antiox12030685)
Supplement: Supplementary file 1 [file antioxidants-12-00685-s001.zip › supplementary file/file S1.pdf]

Widely targeted metabolomics analysis of ALPT aqueous extract

1 Sample preparation and extraction

1. Take out the sample from the -80 °C refrigerator and thaw it on ice , vortex for 10 s to mix well.
2. Take 200 μL of the sample, add it to the 2.0 mL centrifuge tube.
3. Add 200 μL of 70% methanol internal standard extract, vortex for 3 min.
4. Centrifuge at 12000 r/min for 10 min at 4 °C.
5. Pipette the supernatant, filter it with a microporous membrane (0.22 μm), and store it in an injection bottle for LC-MS/MS detection.

2 UPLC Conditions

The sample extracts were analyzed using an UPLC-ESI-MS/MS system (UPLC, ExionLC™ AD , https://sciex.com.cn/; MS, Applied Biosystems 6500 Q TRAP, https://sciex.com.cn/). The analytical conditions were as follows, UPLC: column, Agilent SB-C18 (1.8 μm, 2.1 mm * 100 mm); The mobile phase was consisted of solvent A, pure water with 0.1% formic acid, and solvent B, acetonitrile with 0.1% formic acid. Sample measurements were performed with a gradient program that employed the starting conditions of 95% A, 5% B. Within 9 min, a linear gradient to 5% A, 95% B was programmed, and a composition of 5% A, 95% B was kept for 1 min. Subsequently, a composition of 95% A, 5.0% B was adjusted within 1.1 min and kept for 2.9 min. The flow velocity was set as 0.35 mL per minute; The column oven was set to 40°C; The injection volume was 2 μL. The effluent was alternatively connected to an ESI-triple quadrupole-linear ion trap (QTRAP)-MS.

3 ESI-Q TRAP-MS/MS

The ESI source operation parameters were as follows: source temperature 500°C; ion spray voltage (IS) 5500 V (positive ion mode)/-4500 V (negative ion mode); ion source gas I (GSI), gas II(GSII), curtain gas (CUR) were set at 50, 60, and 25 psi, respectively; the collision-activated dissociation(CAD) was high. QQQ scans were acquired as MRM experiments with collision gas (nitrogen) set to medium. DP(declustering potential) and CE(collision energy) for individual MRM transitions was done with further DP and CE optimization. A specific set of MRM transitions were monitored for each period according to the metabolites eluted within this period.
