# Supplementary figures and images for: Aging-Accelerated Mouse Prone 8 (SAMP8) Mice Experiment and Network Pharmacological Analysis of Aged Liupao Tea Aqueous Extract in Delaying the Decline Changes of the Body
Source: Antioxidants (Basel). 2023 Mar 10;12(3):685. doi: 10.3390/antiox12030685 (PMC10045736; doi:10.3390/antiox12030685)

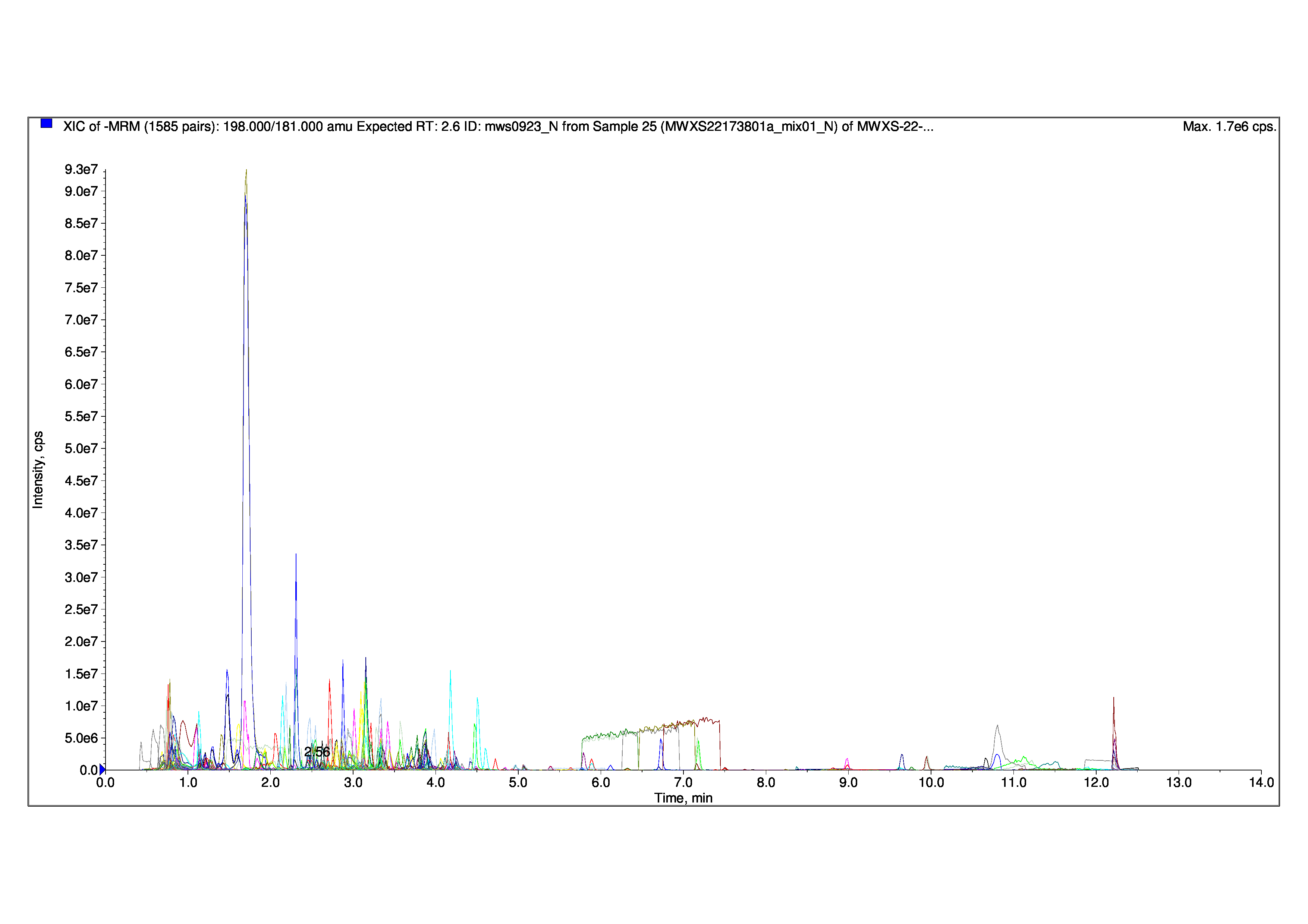

Supplement: Supplementary file 1 [file antioxidants-12-00685-s001.zip › supplementary file/Figure S1/MRM_detection_of_multimodal_maps-N.png]

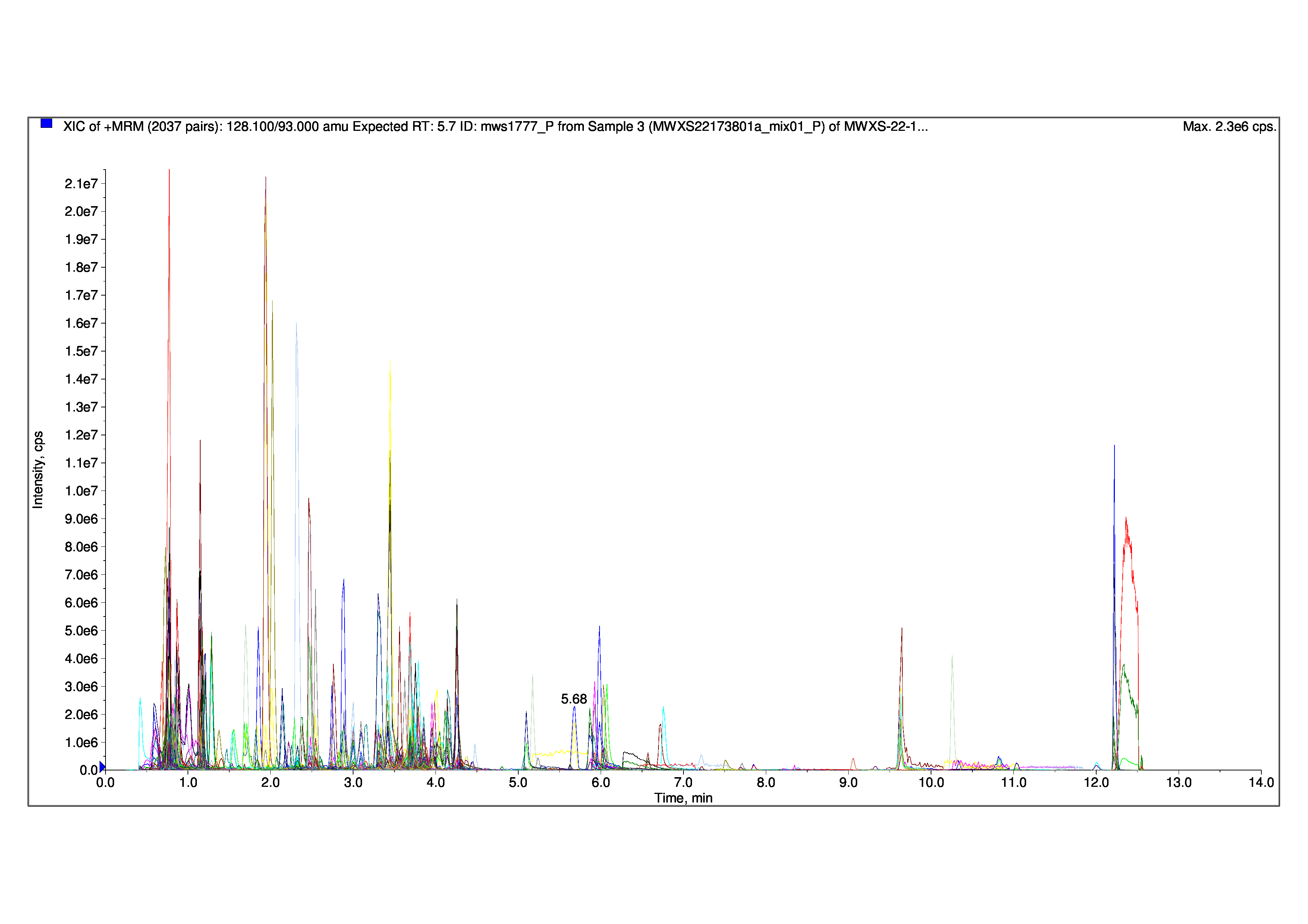

Supplement: Supplementary file 1 [file antioxidants-12-00685-s001.zip › supplementary file/Figure S1/MRM_detection_of_multimodal_maps-P.png]
